# Cyprinid herpesvirus 3 and its evolutionary future as a biological control agent for carp in Australia

**DOI:** 10.1186/s12985-016-0666-4

**Published:** 2016-12-08

**Authors:** Kenneth A. McColl, Agus Sunarto, Edward C. Holmes

**Affiliations:** 1 CSIRO-Australian Animal Health Laboratory, Geelong, VIC 3220 Australia; 2 CSIRO Health and Biosecurity, Geelong, VIC 3220 Australia; 3 Marie Bashir Institute for Infectious Diseases and Biosecurity, Charles Perkins Centre, School of Life and Environmental Sciences and Sydney Medical School, the University of Sydney, Sydney, NSW 2006 Australia

**Keywords:** Biological invasion, *Cyprinus carpio*, Cyprinid herpesvirus 3, Host specificity, Virulence, Transmission, Evolution, Virus

## Abstract

Biological invasions are a major threat to global biodiversity. Australia has experienced many invasive species, with the common carp (*Cyprinus carpio* L.) a prominent example. Cyprinid herpesvirus 3 (CyHV-3) has been proposed as a biological control (biocontrol) agent for invasive carp in Australia. Safety and efficacy are critical factors in assessing the suitability of biocontrol agents, and extensive host-specificity testing suggests that CyHV-3 is safe. Efficacy depends on the relationship between virus transmissibility and virulence. Based on observations from natural outbreaks, as well as the biology of virus-host interactions, we hypothesize that (i) close contact between carp provides the most efficient transmission of virus, (ii) transmission occurs at regular aggregations of carp that favour recrudescence of latent virus, and (iii) the initially high virulence of CyHV-3 will decline following its release in Australia. We also suggest that the evolution of carp resistance to CyHV-3 will likely necessitate the future release of progressively more virulent strains of CyHV-3, and/or an additional broad-scale measure(s) to complement the effect of the virus. If the release of CyHV-3 does go ahead, longitudinal studies are required to track the evolution of a virus-host relationship from its inception, and particularly the complex interplay between transmission, virulence and host resistance.

## Background

Along with climate change and habitat destruction, biological invasions represent one of the great human threats to global biodiversity [1]. Australia has a long and well-documented history of destructive invasive species [2]. In recent years considerable attention has been directed towards common carp (*Cyprinus carpio* L.), which now comprise up to 90% of the fish biomass in parts of the Murray-Darling Basin in south-eastern Australia [3]. These invasive carp have profound impacts on the biodiversity and function of this major and iconic river system, including increased water turbidity, a reduction in submerged vegetation, and changes in the composition of invertebrate communities [3].

Unsurprisingly, the ecological burden imposed by carp has stimulated research into potential control measures. Key among these is the proposed release (in 2018) of cyprinid herpesvirus 3 (CyHV-3) – a double-stranded DNA virus first recognized in the late 1990s [4] – as a biological control agent [5]. Australia is one of the very few localities in which viruses have successfully been employed as biocontrols against vertebrates, involving the release of myxomavirus (MYXV) in the 1950s and rabbit haemorrhagic disease virus (RHDV) in the 1990s to control invasive European rabbits. Here, we argue that as well as its ecological and economic benefits, the release of CyHV-3 will help us to better understand host-pathogen co-evolution at a continental scale.

### The safety of CyHV-3

As it is critical that a biocontrol virus only affects the target host species, the species-specificity of the virus is obviously the most important safety requirement. Extensive host-specificity testing, involving a wide taxonomic range of animals, has been conducted for CyHV-3. Crucially, there is no evidence that CyHV-3 causes clinical, or histological, signs of disease in any non-target species (McColl et al. accepted). Nor is there evidence that the virus even replicates in any of the 14 species of fish, two species each of amphibians and reptiles, and single species of bird, mammal and invertebrate tested [6]. Importantly, representatives of the native Australian taxon most closely-related to common carp (order Siluriformes: salmon catfish, *Neoarius graeffei*; eel-tailed catfish, *Tandanus tandanus*; [7]) were among those fish analysed. The absence of replication in novel hosts, including the closely related native species, seemingly precludes any transient ‘spill-over’ or sustained cross-species transmission [8] occurring with CyHV-3. Interestingly, although cross-species transmission is central to disease emergence [9], host-jumping to species other than lagomorphs has not occurred with either MYXV or RHDV [10], suggesting that there are important constraints to this process [11].

### Evolution of virulence in CyHV-3

Biocontrol viruses need to be both readily transmissible and highly virulent, requirements that are complicated by a possible evolutionary trade-off between these two variables [12]. It is therefore important to ask how might these traits evolve in the case of CyHV-3? Although predictions in this area are inherently difficult with, for example, different levels of virulence seemingly favoured in MYXV and RHDV [10], we hypothesise that close contact between carp is required for efficient transmission of the virus and that this has a number of implications for transmissibility and virulence. The importance of close contact is supported by a number of observations: (i) skin is the major portal of viral entry in carp [13], and both physiological and immunological changes in the skin and surface mucus following infection suggest that skin is also the major site of virus shedding [14]; (ii) CyHV-3 only survives for about three days outside the host [15]; (iii) virus is excreted at low titre and for long periods before the development of clinical disease [16]; (iv) carp are highly sensitive to CyHV-3 infection implying that even transient direct host-to-host contact may allow transmission [17]; and, (v) CyHV-3 likely results in latent infections in survivors [18], thereby allowing repeated opportunities for virus excretion in the face of stress [19]. Together, these observations are consistent with a virus-host relationship that has evolved to facilitate maximum transmissibility during periods of carp aggregation (such as breeding) when fish are also likely stressed and immunosuppressed [20]. These observations then raise a key question: is the transmissibility of CyHV-3 optimized by the evolution of low virulence strains of virus that induce latency in fish? This would enable efficient transmission at those regular periods in the life-cycle when aggregations of infected and uninfected stressed fish lead to recrudescence of latent virus. If true, then it also seems reasonable to predict that the initial high virulence of this virus in *C. carpio* in Australia will gradually decline, mirroring what has happened with MYXV [21], although selection pressures may change as the density of carp declines.

### The evolution of host resistance

Biocontrol viruses will very likely select for the evolution of host resistance, which has been demonstrated in both MYXV and RHDV [10]. It is therefore straightforward to predict that this will also occur following the release of CyHV-3, although what host and viral gene changes will be involved, over what time-scale they will occur, and how mounting resistance will impact virulence evolution are currently impossible to determine. Nevertheless, the inevitability of resistance has already led to the realization that the successful control of carp in Australia will likely require not only the release of successive generations of CyHV-3, each progressively more virulent than its predecessor, but also an additional broad-scale measure to complement the effect of CyHV-3 (for example, ‘daughterless’ carp technology; [22]).

It is also theoretically possible that the use of an imperfectly immunizing vaccine will lead to the evolution of progressively more virulent CyHV-3 strains. A modified-live vaccine has been employed in intensive carp aquaculture for about a decade in some countries [23]. An interesting comparison here is with Marek’s disease virus (MDV), a herpesvirus that is important in the poultry industry. In the 1950s and 1960s, the appearance of virulent MDV strains forced the introduction of Marek’s disease vaccines in 1970 [24]. However, ‘very virulent’ MDV began to appear within ten years, necessitating a next-generation vaccine, followed, in about the same time-frame, by the appearance of ‘very virulent plus’ MDV. The latter resulted in the more widespread use of the CVI988 (Rispens) vaccine that had been in commercial use in the Netherlands since the early 1970s (KA Schat, pers comm). Although this evolutionary trajectory again depends on the exact relationship between virulence and transmissibility [25], if such a similar stepwise selective process does occur in CyHV-3 it may potentially generate highly virulent strains that could be utilized as the next generation of biocontrol viruses.

## Conclusions

To use CyHV-3 as a biocontrol virus for carp in Australia it is critically important to determine the relationship between virulence and transmissibility. Although there are no published mortality figures for the years following a natural outbreak of CyHV-3, observations in wild and farmed populations of carp where CyHV-3 is endemic suggest that, following an initial high mortality, there is a rapid trend toward lower mortalities [26–28]. Although this suggests that the transmissibility of CyHV-3 is favoured by the evolution of low virulence strains, which may also occur after the release of CyHV-3 as biocontrol agent, this relationship could be affected by mounting host resistance and differences in carp population density of wild carp compared with farmed carp. High density aggregations of wild carp are likely to continue (for example, at breeding sites) even in the face of an overall decline in carp population density in the environment at large.

Whatever the nature of the relationship between transmissibility and virulence, the release of CyHV-3 undoubtedly represents a unique and exciting natural experiment that will provide the first opportunity to track, in real time, the co-evolution of both the biocontrol virus and the targeted host. Such a study could lead to major fundamental advances in our understanding of both pathogen evolution and the biological control of invasive pest species.

## Abbreviations

Biocontrol: Biological control
CyHV-3: Cyprinid herpesvirus 3
MDV: Marek’s disease virus
